# Histopathologic Evaluation of Polymer Supports for Pintucci-type Keratoprostheses: An Animal Study

**DOI:** 10.18502/jovr.v14i3.4779

**Published:** 2019-07-18

**Authors:** Saeed Rahmani, Mozhgan Rezaei Kanavi, Mohammad Ali Javadi, Masoumeh Meskinfam Langroudi, Sasha Afsar Aski

**Affiliations:** ^1^Ophthalmic Research Center, Shahid Beheshti University of Medical Sciences, Tehran, Iran; ^2^Department of Optometry, School of Rehabilitation, Shahid Beheshti University of Medical Sciences, Tehran, Iran; ^3^Ocular Tissue Engineering Research Center, Shahid Beheshti University of Medical Sciences, Tehran, Iran; ^4^Department of Chemistry, Lahijan Branch, Islamic Azad University, Lahijan, Iran

**Keywords:** Fibrovascular Tissue Ingrowth, Keratoprosthesis, Pintucci, Polymer

## Abstract

**Purpose:**

To report histopathological findings for different types of polymers proposed as support for a Pintucci-type keratoprosthesis.

**Methods:**

Six polymers, including three types of polyesters (#1-3), one type of polytetrafluoroethylene (PTFE, #4), polyethylene (#5), and expanded polytetrafluoroethylene (ePTFE, #6) were evaluated. Four samples of each material were placed under the orbicularis oculi muscles of 12 rabbits. After five weeks, the samples were removed and evaluated histopathologically. Fibrovascular tissue ingrowths were investigated in terms of tissue penetration depth into the materials (graded as none, mild, moderate, and intense) and fibrovascular ingrowth area at the ultimate level of tissue penetrance. ImageJ software was used to calculate fibrovascular tissue area between the material fibers, and the mean area values were compared between the materials.

**Results:**

Polyester materials #1 and #3 demonstrated intense fibrovascular tissue penetration with a large fibrovascular ingrowth area; no overt tissue ingrowth was observed into material #6. The mean area of penetrated fibrovascular tissues was significantly different between materials (P < 0.001). Materials #2, #4, and #5 showed moderate fibrovascular tissue ingrowth and the area of presented fibrovascular tissue at the paracentral parts of material #4 was significantly smaller than that of materials #1 (P = 0.02) and #3 (P = 0.01).

**Conclusion:**

Two polyester materials that had relatively large pore sizes demonstrated a deep and large area of fibrovascular ingrowth. Given that material #3 is thicker and more consistent than material #1, the former can be used as the appropriate material for supporting the Pintucci-type keratoprosthesis.

##  INTRODUCTION

A keratoprosthesis (KPro) is an artificial or prosthetic cornea made of synthetic materials to restore vision in patients with severe ocular surface diseases.^[1,2]^ The idea of a KPro was introduced in 1779 by Pellier de Quengsy and was then improved structurally and functionally. Since then, various types of KPros have been introduced, among which, Boston (B-Kpro) type I, a modified form of Dohlman-Doan KPro, and osteo-odonto keratoprosthesis (OOKP) are the two types of KPros that are commonly used worldwide.^[1,3]^ Most of the current KPros have a cylinder of polymethylmethacrylate (PMMA) that is a suitable optical material.^[4,5]^ They also have a support (haptic or skirt) which enables a long-lasting integration of the KPro into the body. The support portion of the KPros is the challenging part that has been made from various materials such as Teflon in Cardona Kpro, expanded polytetrafluoroethylene (ePTFE) in Legeais KPro, and alveolar bone in OOKP. In all types of KPros, cellular or tissue ingrowth is pivotal for the support portion of the biointegrable material and plays an important role in the prevention of KPro extrusion.^[1,5,6,7]^

The Boston type I KPro is generally indicated in patients with corneal blindness, who are at high risk of a graft failure, and in patients with severe corneal opacities. An adequate tear secretion is a prerequisite for success after the KPro implantation. However, in patients with corneal blindness and severe dry eyes, such as Stevens-Johnson syndrome (SJS), the ocular cicatrizing pemphigoid (OCP) and chemical burns, OOKP, is the best choice.^[1,7,8,9]^ OOKP has a biologic support and is not applicable for patients that have unsuitable alveolar or tibial bones.^[1,5]^ Alternatively, the Pintucci KPro, a totally synthetic biocompatible material, was developed to fulfill this task.^[10,11]^

Given that the Pintucci KPro is not produced anymore, designing a Pintucci-type KPro (PTKPro) was planned at the Ophthalmic Research Center, Shahid Beheshti University of Medical Sciences, Tehran, Iran. For the supportive skirt of this PTKPro, various types of materials were introduced. In order to evaluate the rate of fibrovascular ingrowth into the introduced materials, this in vivo study was conducted to achieve the proper support for the PTKPro.

##  METHODS

The experiment was conducted according to the Association for Research in Vision and Ophthalmology (ARVO) statement for the use of animals in ophthalmic research and approved by the ethics committee of the Ophthalmic Research Center, Shahid Beheshti University of Medical Sciences, Tehran, Iran.

###  Polymers and Scanning Electron Microscopy

Four disc-shaped samples of six biocompatible and non-degradable materials^[12]^ were provided: material #1, polyester felt of 0.3 mm thickness; material #2, polyester felt of 0.6 mm thickness; material #3, polyester felt of 0.7 mm thickness; material #4, PTFE felt of 0.9 thickness; material #5, polyethylene sheet of 0.4 mm thickness; and material #6, expanded PTFE (ePTFE) felt of 0.6 mm thickness. One sample per material was sent for scanning electron microscopy (SEM) analysis to get the information about the structure of the polymers. The rest of the samples were subjected to plasma sterilization before conducting the animal study.

###  Animals

Twelve female, white, New Zealand rabbits weighing approximately 2 kg were used in this study. Animals were housed with food and water provided ad libitum. Based on the numbers of the materials, rabbits were randomized into six groups. Each material was implanted subcutaneously in four eyes from two animals. Briefly, under general anesthesia with intramuscular 10% ketamine and 2% xylazine, a linear 1.5 cm incision was made through the skin and orbicularis muscle of the lower lids of both eyes. Then, one sample (with 10 mm diameter and of circular shape) of each material was placed under the orbicularis oculi muscle and the incision site was sutured with 7-0 nylon (Supa Medical Devices Co., Karaj, Iran), followed by topical application of tetracycline ointment. Thus, each rabbit received two samples of one type of material. During the operation, artificial tear was used to prevent eye dryness. After five weeks, the incision sites were re-opened and the samples were removed, fixed in 10% formalin, and sent for histopathological examinations. The animals were rehabilitated after re-suturing the incision sites.

###  Histopathological Analysis

After bisecting the removed materials, processing, and embedding into paraffin blocks, thin sections from the peripheral and central aspects of the paraffin-embedded specimens were prepared and stained with hematoxylin and eosin. Five consecutive histologic sections 250 μm apart were prepared from each paraffin block. Stained slides were then examined under a light microscope (BX41, Olympus, Japan), in terms of the presence and depth of penetration of fibrovascular ingrowth into the material in each histologic section. The depth of fibrovascular penetration was then graded as none, mild, moderate, and intense. None was defined when there was no fibrovascular penetration. Mild fibrovascular ingrowth was considered when there was only peripheral involvement. Moderate penetration was defined when the fibrovascular ingrowth was noted at the para-central areas without the involvement of the central parts. In case of involvement of the central parts of the material, the fibrovascular ingrowth was graded as intense. In addition to the depth of fibrovascular penetration, the area of fibrovascular ingrowth for each material was also calculated using the ImageJ software (ImageJ, http://imagej.nih.gov/ij/; provided in the public domain by the National Institutes of Health, Bethesda, MD, USA). For this purpose, three photomicrographs with the same scale bar and from the ultimate level of fibrovascular tissue penetration were captured from each stained slide and the area of the fibrovascular tissue in each photograph was quantified via the ImageJ/Plugins/Macro menu. Mean calculated areas were then compared among the materials using one-way ANOVA and multiple comparisons tests. A P-value < 0.05 was considered statistically significant.

##  RESULTS

During the study, one rabbit that had ePTFE implantation died due to gastroenteritis and was excluded from the study. Overall, 22 samples from six biocolonizable materials were subjected to histopathological examinations.

**Table 1:** Physical characteristics of the examined polymers


** Polymer/Parameter**	**Thickness (mm)**	**Fiber/Fascicle Diameter (μm)**	**Pore Size (μm)**
Material ≠ 1 (Polyester)	0.3	11.38 ± 2.1	60.01 ± 31.08
Material ≠ 2 (Polyester)	0.6	15.36 ± 2.26	32.57 ± 10.88
Material ≠ 3 (Polyester)	0.7	21.79 ± 1.11	72.29 ± 34.12
Material ≠ 4 (PTFE)	0.9	225.5 ± 35.77	167.7 ± 88
Material ≠ 5 (Polyethylene)	0.4	482.34 ± 105.53	132.55 ± 71.9
Material ≠ 6 (ePTFE)	0.6	0.43 ± 0.26	1.4 ± 0.89
	
	
ePTFE, expanded polytetrafluoroethylene; PTFE, polytetrafluoroethylene			

###  Polymers and SEM Findings

**Figure 1:**
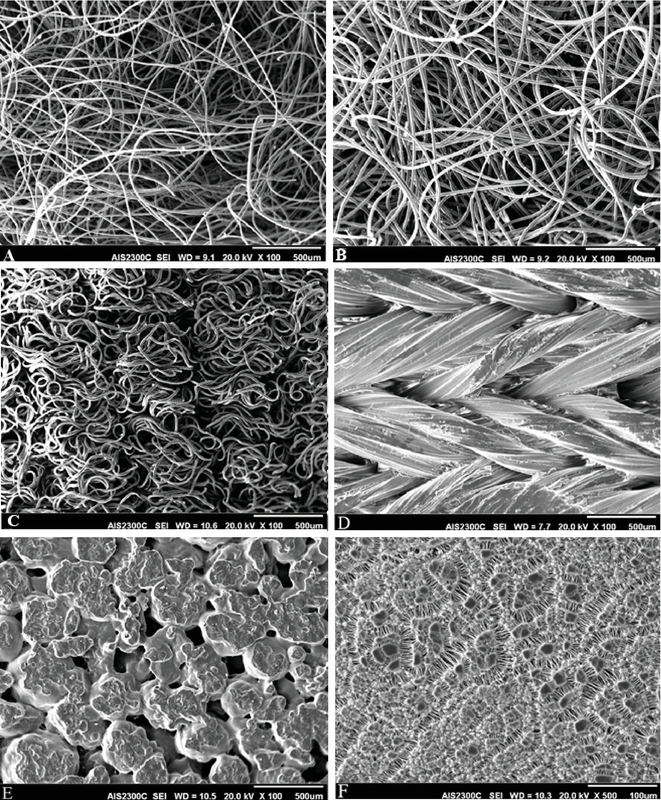
Scanning electron microscopy of examined polymers. (A & B) anisotropic medium composed of randomly dispersed filaments; (C) a number of single fibers compacted with each other in an almost vertical status; (D) woven fascicles of polytetrafluoroethylene consisting of fine microfilaments and disperse coating on PTFE woven fabric so that the whole fiber surface is covered; (E) pores of different shapes and without uniform distribution; (F) open and straight porous structure with an almost uniform slit-like pores in the surface of the expanded polytetrafluoroethylene fabric.

Figure 1 illustrates the SEM images of the materials. The mean values of the pore size of materials #1 to #6 were 60.0 ± 31.0 μm, 32.5 ± 10.8 μm, 72.2 ± 34.1 μm, 167.7 ± 88.0 μm, 132.5 ± 71.9 μm, and 1.4 ± 0.8 μm, respectively [Table 1]. The mean values of fiber diameter in materials #1, #2, #3, and #6 were 11.3 ± 2.1 μm, 15.3 ± 2.2 μm, 21.7 ± 1.1 μm, and 0.4 ± 0.2 μm, respectively. Material #4 was a complex of woven fascicles with a mean diameter of 225.5 ± 35.7 μm, consisting of fine microfibers. Material #5 was presented as confined large fascicles with a diameter of 482.34 ± 105.53 μm containing consolidated fascicles [Figure 1].

###  Histopathological Findings

**Figure 2:**
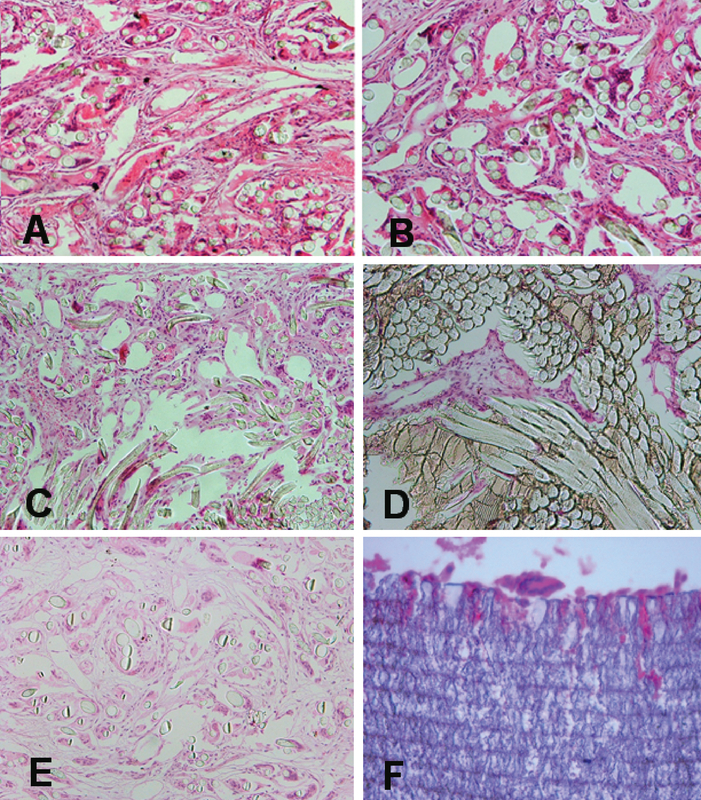
Representative illustrations of fibrovascular ingrowth in various materials. Note the presence of an intense fibrovascular ingrowth into the center of the materials #1 (A) and #3 (B). Materials #2 (C), #4 (D), and #5 (E) revealed a moderate fibrovascular ingrowth up to the paracentral parts of each material. No fibrovascular penetration was noted into material #6 (F). A foreign-body type granulomatous reaction is evident within materials #1 to #5 and around material #6 (H&E, magnification 200×).

**Figure 3:**
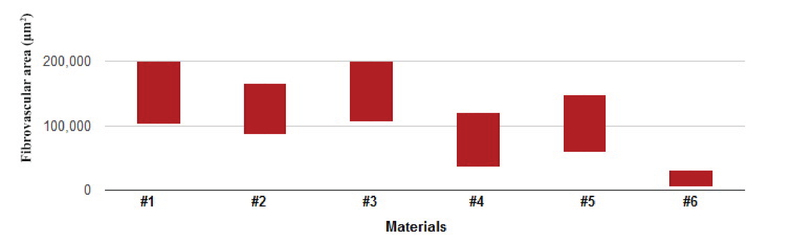
Bar chart of the mean areas of fibrovascular tissue represented in materials #1 to #6, displaying high values for materials #1 and #3 and low values for materials #4 and #6.

Materials #1 and #3 revealed an intense fibrovascular ingrowth at the central parts, with mean areas of 150635.2 ± 48802.5 μm${}^{2}$ and 152472.5 ± 47277.1 μm${}^{2}$, respectively. However, the ePTFE material (#6) did not demonstrate overt fibrovascular penetration and the mean area of fibrovascular tissue at the outermost location of the material was 18098 ± 12963.0 μm${}^{2}$. A moderate fibrovascular ingrowth was observed in the rest of materials [Figure 2]. Mean fibrovascular areas at the paracentral parts of materials #2, #4, and #5 were 125819.0 ± 39651.7 μm${}^{2}$, 77962.5 ± 41939.3 μm${}^{2}$, and 103506.4 ± 44226.9 μm${}^{2}$, respectively. The areas of fibrovascular ingrowth into the examined materials were significantly different between materials (P < 0.001); material #6 demonstrated the least fibrovascular area (P < 0.05), compared to the other five materials, which was limited to the outermost part of the material. The area of fibrovascular ingrowth in material #4 was significantly less than in materials #1 (P = 0.02) and #3 (P = 0.01) [Figure 3]. Although the fibrovascular areas in materials #1 and #3 were larger than those of materials #2 and #5, the difference was not statistically significant. Foreign-body granulomatous reactions composed of epithelioid cells, lymphocytes, and foreign-body type multinucleated giant cells were observed within the fibrovascular ingrowth in specimens #1 to #5 and just around specimen #6. Neutrophils were not evident in the tissue granulomatous reactions and gram-stained slides disclosed no microorganisms.

##  DISCUSSION

In addition to stability, the KPro support functions as a barrier to microbial penetration and prevents extrusion of the KPro. In patients with corneal blindness and severe dry eye, a KPro with biologic support, such as the OOKP, is the best choice, in which the support is provided from a bone autograft and is well tolerated by the patient.^[1,13]^ However, in some instances where there is no suitable bone, the use of a biointegrable KPro may be considered. The biointegrable supports integrate into the implanted site via fibrovascular ingrowth and increases the KPro stability. Polyester (Dacron) as a porous material has good physical and chemical specifications. In contrast to some other materials, such as metals and plastics, Dacron is a non-degradable, soft, pliable, and inert material that has been successfully used as a support for Girard and Pintucci KPros.^[10,11][14]^ Although some biocompatible porous materials, such as hydroxyapatite and bio-active glass, have been examined experimentally as substitutes for the bony support of OOKP, the safety and efficacy of these materials in human subjects have not been determined.^[15,16,17,18]^

The present in-vivo study investigated the rate of fibrovascular ingrowth into the various types of biocompatible and non-degradable materials to obtain a proper material for supporting the designed PTKPro. A high degree of penetration, together with a large area of fibrovascular ingrowth at the central region, was observed in the polyester materials that had similar pore sizes for tissue penetration. The fibrovascular ingrowth was very limited in the ePTFE material that had the smallest pore size. Comparisons of the materials in terms of fiber diameter and pore size revealed that the type of the material used and the pore size were critical factors for the induction of fibrovascular ingrowth.^[19]^ For instance, amongst the three polyesters examined in our study, materials #1 and #3, which had a greater pore size (60 μm and 72 μm, respectively) than that of material #2 (33 μm) [Table 1], induced a higher degree and a larger area of fibrovascular tissue penetration than material #2. As for the PTFE (#4) and polyethylene (#5) materials, it seems that the high thickness of the fascicles (226 μm and 482 μm, respectively) may reverse the effectiveness of the large pore size of these materials for tissue ingrowth. The presence of consolidated fascicles in material #5 may have an additional inverse effect on tissue penetration. In the current study, the fibrovascular area at the paracentral parts of material #4 was shown to be significantly less than that of materials #1 and #3. Additionally, fibrovascular ingrowth in material #5 was not observed beyond the paracentral sections of the material and the area of fibrovascular tissue at the paracentral region was smaller than that of materials #1 and #3, although the difference was not statistically significant.

In our series, the polyester material of 0.7 mm thickness revealed the best results for fibrovascular ingrowth. These results were comparable with those reported by Pintucci et al,^[11]^ in which polyester (Dacron) fabric of 0.6 mm thickness was preferred for tissue penetration to the polyester fabrics of 0.25 mm and 1.4 mm thickness. In addition to more consistency, it seems that polyesters of 0.6-0.7 mm thickness may provide better handling during KPro implantation than the other examined thicknesses. Our experimental setting was similar to the standard protocol, recommended by Pintucci et al, for KPro procedure in humans^[11]^ and the samples were maintained subcutaneously for approximately five weeks.

In soft KPros, which are recently implanted as corneal interlamellar KPros, poly-2-hydroxyethyl methacrylate, PTFE, or ePTFE are the materials that have been used for the support of the KPro. The main histopathological outcomes for biointegration of these KPros were the invasion of keratocytes or corneal tissue ingrowth into the KPro supports.^[6,20,21,22]^ For instance, in an experimental study by Caldwell, the implanted ePTFE material within the corneal stroma of a rabbit was the best material for the induction of fibrovascular ingrowth as compared with Dacron or PTFE. The ePTFE materials implemented in the Caldwell study had different pore sizes (30 μm, 60 μm, and 90 μm); of these, the ePTFE of 60 μm pore size was preferred for using as a KPro support.^[6]^ It is noteworthy to highlight that the pore size of ePTFE can affect the cellular ingrowth into the material. For instance, in the studies by Legeais et al and Liang et al, pore sizes of 45 to 50 μm^[20,21]^ were reported as the best pore sizes for cell ingrowth into the ePTFE. However, in another type of corneal interlamellar KPro, the so-called Seoul-type KPro, out of four types of porous polymers examined as the support of the KPro, those of pore sizes larger than 30 μm allowed fibroblast invasion into the support material.^[23]^ The ePTFE used in our series, when compared with the ones used in the aforementioned studies, had a smaller pore size (1.4 μm), which may explain the poor penetration of fibrovascular tissue into the sample in the current study.

The presence of fibrovascular tissue, together with a foreign-body granulomatous inflammation within specimens #1 to #5 in our series, was indicative of an advanced healing process in which the porous specimens were penetrated by the ingrowth of vascularized connective tissue. These biointegration properties of the colonized implanted specimens may behave further as an “autotissue", when they are subsequently transplanted as the KPro supports.

In conclusion, our study demonstrates that the polyester materials of 60 μm and 72 μm pore sizes show the best fibrovascular ingrowth, in terms of the depth of the penetration and the area of penetrated fibrovascular tissue. Considering that the 0.7 mm thickness of the polyester materials is similar to that used by Pintucci et al, providing easy handling during surgery, with optimum support and consistency, they can be considered as the materials of choice for the PTKPro support.

##  Financial Support and Sponsorship

Nil.

##  Conflicts of Interest

There are no conflicts of interest.

## References

[1] Avadhanam V. S., Smith H. E., Liu C. (2015). Keratoprostheses for corneal blindness: A review of contemporary devices. *Clinical Ophthalmology*.

[2] Akpek E. K., Alkharashi M., Hwang F. S., Ng S. M., Lindsley K. (2014). Artificial corneas versus donor corneas for repeat corneal transplants. *Cochrane Database of Systematic Reviews*.

[3] Salvador-Culla B., Kolovou PE. (2016). Keratoprosthesis: A review of recent advances in the field. *J Funct Biomater*.

[4] Vijayasekaran S., Robertson T., Hicks C., Hirst L. (2005). Histopathology of long-term cardona keratoprosthesis: A case report. *Cornea*.

[5] Hille K., Hille A., Ruprecht K. W. (2006). Medium term results in keratoprostheses with biocompatible and biological haptic. *Graefe's Archive for Clinical and Experimental Ophthalmology*.

[6] Caldwell DR. (1997). *The soft keratoprosthesis. Trans Am Ophthalmol Soc*.

[7] Gomaa A., Comyn O., Liu C. (2010). Keratoprostheses in clinical practice - a review. Clin Exp Ophthalmol. *Liu C. Keratoprostheses in clinical practice - a review. Clin Exp Ophthalmol*.

[8] Brown C. R., Wagoner M. D., Welder J. D., Cohen A. W., Goins K. M., Greiner M. A., Kitzmann A. S. (2014). Boston keratoprosthesis type 1 for herpes simplex and herpes zoster keratopathy. *Cornea*.

[9] Chang H.-Y. P., Luo Z. K., Chodosh J., Dohlman C. H., Colby K. A. (2015). Primary implantation of type i Boston keratoprosthesis in nonautoimmune corneal diseases. *Cornea*.

[10] Pintucci S., Pintucci F., Cecconi M., Caiazza S. (1995). New Dacron tissue colonisable keratoprosthesis: Clinical experience. *British Journal of Ophthalmology*.

[11] Pintucci S., Perilli R., Formisano G., Caiazza S. (2001). Influence of Dacron tissue thickness on the performance of the Pintucci biointegrable keratoprosthesis: An in vitro and in vivo study. *Cornea*.

[12] Shastri VP. (2003). *Non-degradable biocompatible polymers in medicine: past, present and future. Curr Pharma Biotechnol*.

[13] Hille K. (2018). Long-term outcome of keratoprosthesis with biological support. *Der Ophthalmologe*.

[14] Girard LJ., Hawkins RS., Nieves R., Borodofsky T., Grant C. (1977). *Keratoprosthesis, a 12-year follow-up. T Sec Ophthalmol Am Acad Ophthalmol Otolaryngol*.

[15] Huhtinen R., Sandeman S., Rose S., Fok E., Howell C., Froberg L. (2013). Examining porous bio-active glass as a potential osteo-odonto-keratoprosthetic skirt material. *J Mater Sci Mater Med*.

[16] Ciolino JB., Dohlman CH. (2009). Biologic keratoprosthesis materials. Int Ophthalmol Clin. *Dohlman CH. Biologic keratoprosthesis materials. Int Ophthalmol Clin*.

[17] Tan XW., Thompson B., Konstantopoulos A., Goh TW., Setiawan M., Yam GH. (2015). *Application of graphene as candidate biomaterial for synthetic keratoprosthesis skirt. Invest Ophthalmol Vis Sci*.

[18] Mehta J. S., Futter C. E., Sandeman S. R., Faragher R. G. A. F., Hing K. A., Tanner K. E., Allan B. D. S. (2005). Hydroxyapatite promotes superior keratocyte adhesion and proliferation in comparison with current keratoprosthesis skirt materials. *British Journal of Ophthalmology*.

[19] Wu X. Y., Tsuk A., Leibowitz H. M., Trinkaus-Randall V. (1998). In vivo comparison of three different porous materials intended for use in a keratoprosthesis. *British Journal of Ophthalmology*.

[20] Liang D., Chen J., Li Y., Lin J., Chen Z. (1999). Expanded polytetrafluoroethylene with different pore diameter for keratoprosthesis cell ingrowth and corneal metabolism. *Yan ke xue bao = Eye science / "Yan ke xue bao" bian ji bu*.

[21] Legeais J.-M., Renard G., Parel J.-M., Serdarevic O., Mei-Mui M., Pouliquen Y. (1994). Expanded Fluorocarbon for Keratoprosthesis Cellular Ingrowth and Transparency. *Experimental Eye Research*.

[22] Chirila T. V. (2001). An overview of the development of artificial corneas with porous skirts and the use of PHEMA for such an application. *Biomaterials*.

[23] Kim M. K., Lee J. L., Wee W. R., Lee J. H. (2002). Comparative experiments for in vivo fibroplasia and biological stability of four porous polymers intended for use in the Seoul-type keratoprosthesis. *British Journal of Ophthalmology*.

